# Hydatidiform Moles: The Contribution of Ancillary Techniques in Refining Their Histopathological Diagnosis

**DOI:** 10.3390/ijms27010142

**Published:** 2025-12-23

**Authors:** Teodora Ana Balan, Raluca Anca Balan, Cornelia Amalinei, Simona Eliza Giușcă, Irina-Draga Căruntu

**Affiliations:** 1 Grigore T. Popa University of Medicine and Pharmacy Iasi, Department of Morphofunctional Sciences I, 16 University Street, 700115 Iasi, Romania; balan.teodora-ana@d.umfiasi.ro (T.A.B.); cornelia.amalinei@umfiasi.ro (C.A.); simonaelizagiusca@gmail.com (S.E.G.); irina.caruntu@umfiasi.ro (I.-D.C.); 2 Pathology Department, “Elena Doamna” Clinical Hospital of Obstetrics and Gynecology, 49 Elena Doamna Street, 700398 Iasi, Romania; 3 Pathology Department, Institute of Forensic Medicine, 700455 Iasi, Romania; 4 Pathology Department, “Dr. C. I. Parhon” Clinical Hospital, 50 Carol I Boulevard, 700503 Iasi, Romania; 5 Romanian Medical Science Academy, 1 I. C. Bratianu Boulevard, 030171 Bucharest, Romania

**Keywords:** hydatidiform moles, immunohistochemistry, gestational trophoblastic disease

## Abstract

A hydatidiform mole (HM) is the most common form of gestational trophoblastic disease (GTD). Differentiating hydatidiform moles (HMs) from non-molar pregnancies and distinguishing complete HMs (CHMs) from partial HMs (PHMs) remains challenging due to overlapping morphological features and a high rate of misclassification. This study aimed to evaluate reliable immunohistochemical markers for improving diagnostic accuracy and addressing the limitations of current molecular techniques. We retrospectively analyzed 64 cases of HMs and hydropic abortions (HAs), diagnosed in women aged 17–36 years between 2010 and 2024, at the Pathology Department of “Elena Doamna” Clinical Hospital, Iași, Romania. Routine histology was supplemented with immunohistochemistry (IHC) using p57, Ki-67, β-hCG, and E-cadherin, with semiquantitative immunoscores applied. Histology revealed 38 PHMs (59.37%), 16 CHMs (23.88%), and 10 HAs (15.62%). p57 was positive in 100% of PHMs and HAs but only in 18% of CHMs. Ki-67 expression was predominantly strong in CHMs, variable in PHMs, and weak in all HAs. β-hCG showed the highest expression in CHMs, followed by PHMs and HAs, while E-cadherin was strongest in HAs. Morphological features alone are insufficient for HM diagnosis; thus, ancillary techniques like p57 IHC and DNA genotyping are crucial to differentiate complete, partial moles, and non-molar specimens by revealing unique genetic patterns, especially p57 absence in CHMs and ploidy/parental origin in PHMs. In this context, an algorithmic approach integrating histology, immunohistochemistry, and genotyping reduces interobserver variability and refines diagnostic precision.

## 1. Introduction

Gestational trophoblastic disease (GTD) is a group of tumors originating from the placenta, with the most common type being the hydatidiform mole [1]. Its main etiological factors involve maternal age, ethnicity, and genetic aspects [1]. However, the etiology of gestational trophoblastic tumors arising from a normal pregnancy is practically still unknown [1].

Hydatidiform moles (HMs) are a gestational complication, occurring in approximately 0.5 to 1 per 1000 pregnancies in Western countries, being significantly more common in the Far East, affecting 10–12 per 1000 pregnancies in countries like Indonesia and India [1,2]. In the Egyptian population, the incidence of GTD is 6.6 per 1000 live births, a rate notably higher than that seen in Western populations [2]. A 2024 retrospective study found differences in rates and mole type between ethnic groups, with an overall risk of 22 per 10,000 live births in that cohort and variable proportions of complete versus partial moles by ethnicity and age [3]. Although further research is needed to fully understand the epidemiology of these moles, this elevated frequency in Asia compared to other regions may stem from genetic variations within ethnic groups. Geographic patterns also indicate that molar pregnancies are more common in reproductive age extremes, like older maternal age groups (>35 years) and in adolescent pregnancies [1]. The risk notably rises, increasing 5-fold for women over 40 and 2.5-fold for those over 35 [1,4]. Moreover, the chance of recurrent molar pregnancies is greater than that of a single occurrence, along with a higher risk of other gestational trophoblastic diseases, such as choriocarcinoma [4]. Multiple recent reviews or cohort studies report that a complete hydatidiform mole (CHM) has substantially higher risk of post-molar gestational trophoblastic neoplasia (commonly about 15–20% in many recent series), while a partial hydatidiform mole (PHM) typically has a much lower risk (roughly 0.5–5%, with some systematic reviews indicating <1% in many datasets) [5,6,7].

An HM is further classified into two types—partial hydatidiform mole (PHM) and complete hydatidiform mole (CHM)—based on their morphological, genetic, and clinical characteristics. Morphologically, hydropic abortion (HA) can resemble a HM, and despite clear histopathologic criteria, distinguishing HAs from HMs or differentiating CHMs from PHMs remains challenging in clinical settings [8]. This distinction is important for treatment and prognosis, as the risk of developing persistent GTD varies with the type (10–30% for CHMs and 1–7% for PHMs) [9].

Differentiating HMs from non-molar pregnancies and distinguishing CHMs from PHMs is difficult due to overlapping morphological features, especially in early pregnancy. Previous studies have shown low agreement among pathologists and a high rate of misclassification [10,11,12]. Advances in genetic and molecular techniques, including flow cytometry (FC), digital image analysis, and p57 immunostaining, now support confirmatory diagnosis of HMs [9]. FC rapidly analyzes DNA ploidy in a large number of cells, aiding particularly in PHM diagnosis due to its androgenic triploid chromosomal composition. However, FC cannot reliably distinguish HA from a CHM, as both often exhibit diploid or near-diploid chromosomal profiles. FC also remains costly and requires specialized facilities and skilled operators, limiting its routine use [13].

p57 immunostaining targets the *p57KIP2* gene, a maternally expressed and paternally imprinted gene, whose expression is absent in CHMs but present in PHMs and normal trophoblasts. This method is simple and cost effective for confirming CHMs, though it does not differentiate PHMs from non-molar pregnancies [2,10].

Cadherins play a critical role in mediating cell-to-cell adhesion in epithelial tissues and regulating trophoblast cell behavior during placental development. E-cadherin expression, observed on the villous cytotrophoblast cell membrane, is a key marker for identifying PHMs. Studies show higher E-cadherin immunoreactivity in PHMs compared to CHMs and in HAs compared to PHMs, making a combination of Ki-67 and E-cadherin immunoreactivity an effective differentiator of PHMs from non-molar pregnancies [14].

Human Chorionic Gonadotrophin (hCG), a hormone secreted by trophoblastic cells, which is present in early embryonic development, shows cytoplasmic stain, relatively specific for choriocarcinoma or syncytiotrophoblasts. The literature revealed that a CHM presents strong expression of β-hCG, and a PHM presents weak expression of β-hCG, while choriocarcinoma shows strong immunohistochemical expression of β-hCG [15].

The Ki-67 protein is expressed during all active phases of the cell cycle (G1, S, G2, and mitosis) but is absent in quiescent or resting cells (G0). This makes it an excellent marker for assessing tissue proliferation and, therefore, a valuable tool for studying the biological behavior of gestational trophoblastic diseases [16].

Current research aims to find reliable markers for distinguishing and classifying HMs, addressing the limitations of current molecular techniques. To the best of our knowledge, although there is research evaluating the immunoexpression of different molecules to diagnose and understand the pathogenesis of HMs, there is no other published study that simultaneously examines this panel of markers in hydatidiform moles. In this regard, our study was carried out to assess the expression patterns of p57, Ki-67, E-cadherin, and β-hCG in HAs, PHMs, and CHMs and to evaluate the value of these immunohistochemical markers for improving diagnostic accuracy.

Although p57, E-cadherin, β-hCG, and Ki-67 have each been studied individually or combined in complete hydatidiform moles (CHMs), partial hydatidiform moles (PHMs), and hydropic abortions (HAs), reliance on a single marker is insufficient for consistently accurate classification because these lesions differ across genetic origin, trophoblastic differentiation, proliferative activity, and functional hormone production. A combined, simultaneous assessment allows for the integration of these complementary biological dimensions and reduces diagnostic ambiguity.

Considering these aspects, we hypothesize that the combined immunohistochemical evaluation of p57, E-cadherin, β-hCG, and Ki-67 provides superior discriminatory power for differentiating CHMs, PHMs, and HAs than any marker assessed individually, due to the fact that these markers interrogate distinct but complementary biological processes whose integrated expression patterns more accurately reflect the underlying pathogenesis of each entity. Moreover, a structured algorithm that combines histologic assessment with immunohistochemical analysis and DNA genotyping for difficult cases minimizes interobserver variability and enhances diagnostic accuracy.

## 2. Results

### 2.1. Major Findings on the Immunoexpression of the Studied Markers

**Figure 1:**
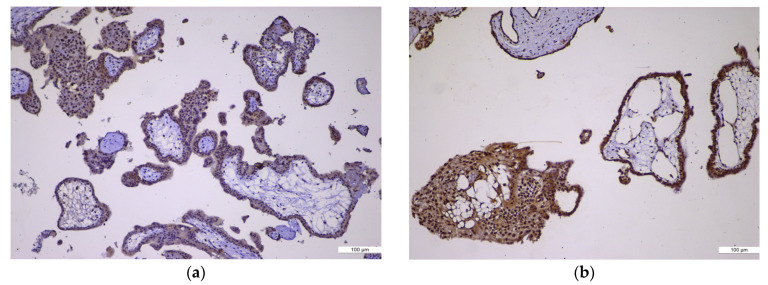
p57 immunoexpression. (**a**) p57 immunoexpression in CHM, ×10: negative in villous cytotrophoblast and stromal cells (absent, no maternal genome); (**b**) p57 immunoexpression in PHM, ×10: positive in villous cytotrophoblast and stromal cells (nuclear staining present).

Among the 16 cases of complete hydatidiform moles (CHMs), 9 cases (57%) showed no p57 immunoexpression, 4 cases (25%) exhibited equivocal expression, and 3 cases (18%) were discordant positive for p57. In contrast, all 38 cases of partial hydatidiform moles (PHMs) and all 10 cases of hydropic abortion (HA) demonstrated positive p57 expression (100%) (Table 1, Figure 1a,b).

**Figure 2:**
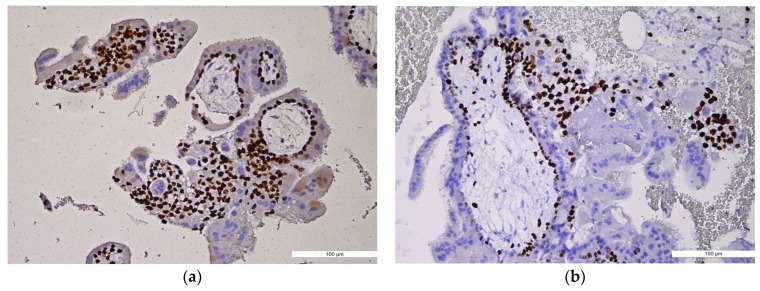
Ki-67 immunoexpression. (**a**) Ki-67 immunoexpression in CHM, ×10: high index, diffuse and strong trophoblastic staining (trophoblastic proliferation); (**b**) Ki-67 immunoexpression in PHM, ×10: moderate, patchy trophoblastic staining.

Regarding Ki-67 expression in CHM cases, two cases (12.5%) showed weak immunostaining, eight cases (50%) showed moderate staining, and six cases (37.5%) exhibited strong immunoexpression. In the PHM group, 21 cases (57.8%) showed weak Ki-67 expression, 9 cases (23.7%) had moderate staining, and 4 cases (10.5%) showed strong immunoexpression. All HA cases (100%) demonstrated weak Ki-67 immunostaining (Table 2, Figure 2a,b).

**Figure 3:**
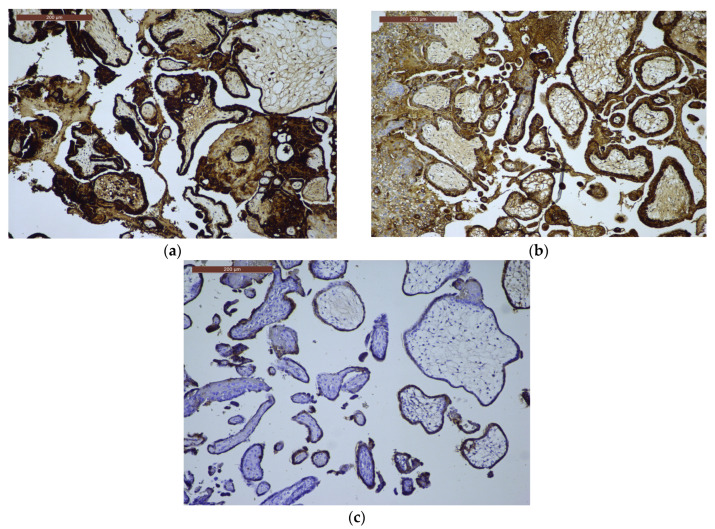
β-hCG immunoexpression. (**a**) β-hCG immunoexpression in CHM, ×10: strong, diffuse trophoblastic proliferation; (**b**) β-hCG immunoexpression in PHM, ×10: variable, moderate trophoblastic staining; (**c**) β-hCG immunoexpression in HA, ×10: weak and focal trophoblastic expression.

For β-hCG immunoscoring, 10 CHM cases (62.5%) had a score of 12, 5 cases (31.25%) scored 9, and 1 case (6.25%) showed a low score of 2, which was considered equivocal. Among PHM cases, 36 (94.7%) scored 6, and 2 cases (5.3%) scored 4. In the HA group, two cases (20%) scored 2, four cases (40%) scored 1, and four cases (40%) showed no expression (Table 3, Figure 3a–c).

**Figure 4:**
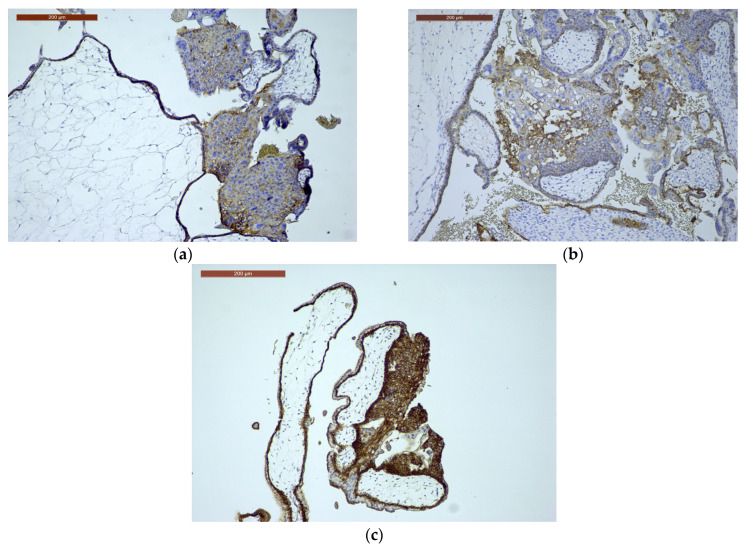
E-cadherin immunoexpression. (**a**) E-cadherin immunoexpression in CHM, ×10: often reduced/aberrant membranous trophoblastic expression (disordered trophoblast); (**b**) E-cadherin immunoexpression in PHM, ×10: relatively preserved membranous trophoblastic staining; (**c**) E-cadherin immunoexpression in HA, ×10: preserved, normal membranous trophoblastic staining.

E-cadherin immunoscores in CHMs were distributed as follows: two cases (12.5%) scored 9, seven cases (43.75%) scored 6, and seven cases (43.75%) scored 2. In PHM cases, 2 (5.3%) had a score of 12, 8 (21.1%) scored 8, 22 (57.9%) scored 6, and 6 (15.8%) scored 4. Among the HA cases, seven (70%) showed a score of 12, two (20%) scored 9, and one case (10%) had a score of 8 (Table 4, Figure 4a–c).

### 2.2. Marker-Specific Performance

The immunohistochemical markers showed significant differences between CHMs, PHMs, and HAs (Table 5).

p57 IHC demonstrated the highest diagnostic specificity for CHMs. Loss of nuclear expression was identified in 57% of CHMs, with 25% showing equivocal staining and 18% discordant positivity, whereas all PHMs and HAs were positive. The association between p57 negativity and CHMs was highly significant (χ^2^ = 26.94, *p* = 2.1 × 10^−7^; Fisher’s exact *p* < 2 × 10^−7^). Excluding equivocal cases, the marker achieved 75% sensitivity, 100% specificity, and 100% PPV (NPV 94.1%). Although ROC analysis is less informative for binary markers, the effective AUC was consistent with excellent discrimination.

β-hCG immunostaining showed the most pronounced quantitative separation between groups (CHM ≫ PHM > HA), with highly significant results (χ^2^ = 121.1, *p* = 3.8 × 10^−20^; Kruskal–Wallis *p* = 4.3 × 10^−12^). Post hoc Mann–Whitney U tests were significant for all pairwise comparisons (*p* < 0.001). ROC analysis demonstrated excellent diagnostic performance (AUC = 0.95). At a threshold of ≥9, β-hCG yielded 93.8% sensitivity and 100% specificity, confirming its role as the most reliable quantitative discriminator for CHMs.

Ki-67 expression reflected a clear proliferative gradient (CHM ≫ PHM > HA), with significant intergroup variation (χ^2^ = 21.1, *p* = 0.0003; Kruskal–Wallis *p* = 3.9 × 10^−5^). Mann–Whitney U testing confirmed significant differences between CHMs and PHMs (*p* = 0.0009) and CHMs and HAs (*p* < 0.0001), while PHMs vs. HAs was borderline significant (*p* = 0.027). ROC analysis indicated good discriminatory power (AUC = 0.81), with 87.5% sensitivity and 71.1% specificity at a moderate/strong expression threshold, supporting its role as a valuable adjunct marker.

E-cadherin immunoexpression was lowest in CHMs, intermediate in PHMs, and highest in HAs, with significant intergroup differences (χ^2^ = 67.7, *p* = 1.3 × 10^−10^; Kruskal–Wallis *p* = 1.1 × 10^−6^). Pairwise Mann–Whitney tests were significant for CHM vs. HA (*p* < 0.0002) and PHM vs. HA (*p* < 0.00001) but not for CHM vs. PHM (*p* = 0.06). ROC analysis demonstrated limited diagnostic discriminative capacity (AUC = 0.25), with 87.5% sensitivity and 41.7% specificity at a ≤6 cut-off.

The low AUC observed for E-cadherin reflects inverse discriminatory behavior, whereby lower immunoscores are associated with CHMs, and higher expression is observed in PHMs and HAs. This finding is consistent with the biological role of E-cadherin as a cell–cell adhesion molecule, whose expression is reduced in CHMs due to abnormal trophoblastic differentiation and disrupted villous architecture. Because ROC curves were constructed using increasing immunoscore values as indicators of CHM positivity, this inverse relationship resulted in an AUC below 0.5. Conceptually, inversion of the scoring direction would yield a complementary AUC indicative of fair discrimination; however, the original orientation was retained for methodological consistency across markers.

Thus, despite statistically significant group differences, E-cadherin demonstrates limited practical diagnostic utility compared with p57 and β-hCG, and its primary value lies in highlighting pathophysiological differences rather than serving as a standalone classifier.

**Figure 5:**
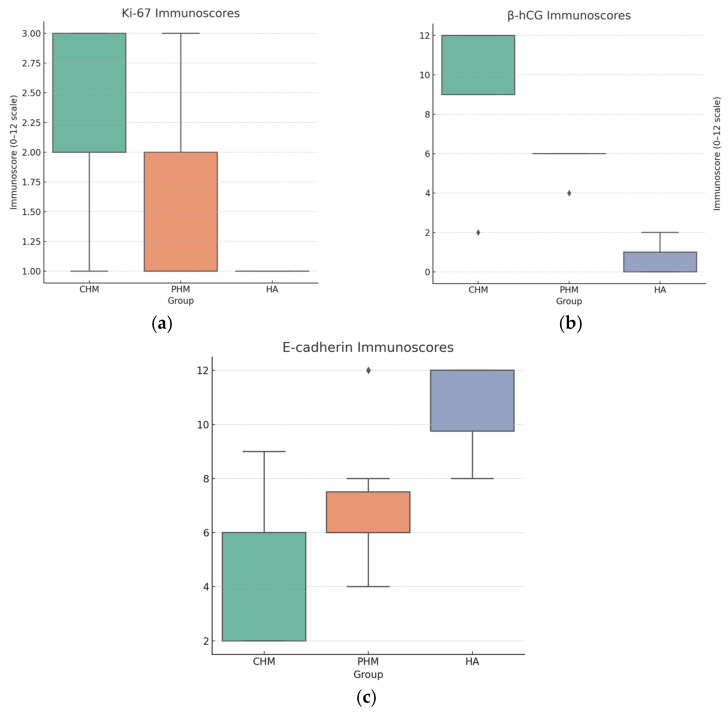
Box plots of immunoscores. (**a**) Ki-67: CHM shows higher scores than PHM and HA, consistent with increased proliferative activity; (**b**) β-hCG: CHM has the highest immunoscores, PHM intermediate, and HA lowest; (**c**) E-cadherin: HA tends to have the highest scores and CHM the lowest, consistent with semiquantitative observations.

**Figure 6:**
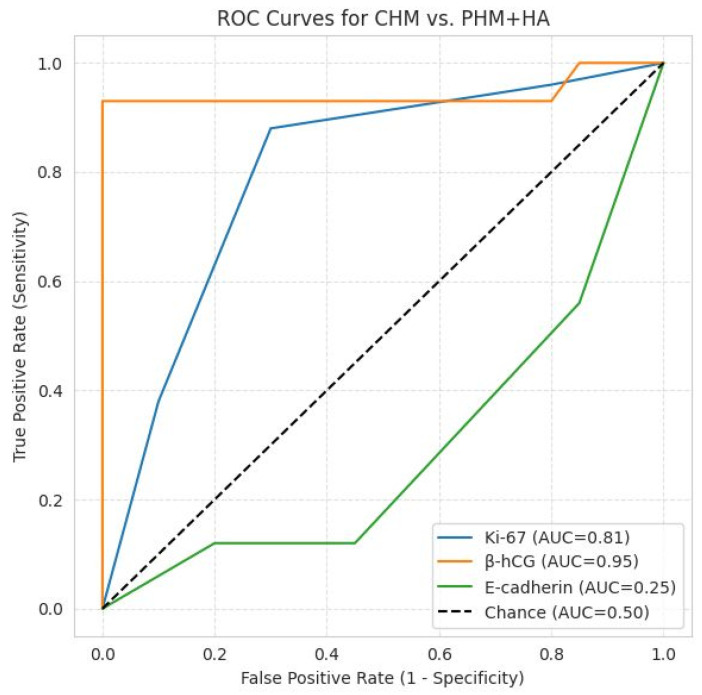
ROC curves for CHM vs. PHM + HA. β-hCG: AUC = 0.95, excellent discriminator; Ki-67: AUC = 0.81, good discriminator; E-cadherin: AUC = 0.25, inverse discrimination, as lower immunoscores are associated with CHM and higher expression with PHM and HA; the diagonal line represents random chance (AUC = 0.5).

ROC analysis provided a comprehensive assessment of marker performance across all thresholds. The β-hCG curve approached the upper-left corner, indicating excellent accuracy, while Ki-67 showed good but moderate discriminative ability. In contrast, the E-cadherin curve lay near or below the reference line, reflecting inverse discrimination. p57, although binary, functionally corresponds to a high-performing classifier due to its absolute specificity for CHMs when absent. Collectively, these findings support an integrated diagnostic approach in which p57 and β-hCG serve as the most reliable markers, Ki-67 provides supportive proliferative information, and E-cadherin contributes primarily to biological characterization but little to diagnostic accuracy (Figure 5a–c and Figure 6).

## 3. Discussion

Molar gestations include a spectrum of trophoblastic disorders that require accurate diagnosis to guide appropriate clinical management, especially for evaluating the risk of persistent gestational trophoblastic disease. A hydatidiform mole (molar pregnancy) (HM) is a gestation-related lesion in which abnormal fertilization produces an aberrant placenta, characterized by variable hydropic swelling of chorionic villi and trophoblastic proliferation, resulting in a non-viable pregnancy, with duplication of the paternal genome, which may be either a complete hydatidiform mole (CHM) with an absent maternal genome or a partial hydatidiform mole (PHM) with a present maternal genome and which may progress to persistent gestational trophoblastic neoplasia (GTN) [17,18].

### 3.1. Ancillary Technique Framework

The present study underscores the imperative role of these ancillary techniques, particularly IHC, in the nuanced differential diagnosis of HMs and HAs. Traditional histopathological evaluation, though foundational, remains hampered by substantial interobserver variability and overlapping morphological hallmarks between CHMs, PHMs, and HAs, especially in early-stage lesions [19,20]. Immunohistochemical techniques can also reduce both turnaround time and cost, which is especially beneficial in low-resource settings [21]. However, according to the literature data, molecular genotyping (short tandem repeat/DNA ploidy analysis) is the true gold standard for classifying hydatidiform moles (CHM vs. PHM vs. non-molar hydropic abortion) [19,20].

To the best of our knowledge, there is no other study that assesses theimmunohistochemical expression of p57, Ki-67, E-cadherin, and β-hCG in differentiating the types of hydatidiform moles versus hydropic abortion. The rationale for choosing these markers was based on the fact that the current literature supports a morphology-first approach supplemented by p57 IHC as the primary triage test [21], with Ki-67 and E-cadherin used as adjuncts to help separate partial mole (PHM), complete mole (CHM), and hydropic non-molar conceptions; although with limited discriminatory value, β-hCG immunoexpression can also highlight trophoblastic cell activity [2,19,22,23].

### 3.2. Features of p57 Immunoexpression

*p57 (CDKN1C)* represents a maternally expressed (paternally imprinted) gene product. Because CHMs are typically androgenetic (absent maternal genome), the villous cytotrophoblast and villous stromal cells lack p57 immunoreactivity, while PHMs and non-molar hydropic abortions retain maternal alleles and, hence, are p57-positive. This makes p57 the single most useful immunohistochemical triage marker. However, rare exceptions (biparental CHM, retained maternal chromosome 11, and mosaicism) and technical pitfalls exist; therefore, p57 negative is strongly supportive of CHMs, but positive p57 does not exclude the need for further workup when morphology is ambiguous [19,22].

Among the markers used in differentiating the types of hydatidiform moles, p57, a maternally expressed and paternally imprinted protein encoded by *CDKN1C* (*Cyclin Dependent Kinase Inhibitor 1C*) gene on chromosome 11p15, emerged as the most robust discriminant. Our findings, 18% p57 positivity in CHMs versus 100% in PHMs and HAs, mirror published evidence that CHMs typically lack maternal genomic contribution and consistently lose p57 expression, whereas both PHMs and HAs retain it [24,25,26,27]. A meta-analysis reported that the immunohistochemical marker p57 has a sensitivity of approximately 98% and specificity of 62% for CHM diagnosis when compared to genotyping [20]. In a large prospective series of 2217 cases, 99.8% of immunostained CHMs were p57 negative, and 99% of PHMs were p57 positive, underscoring its great diagnostic reliability [28].

Complete hydatidiform moles may occasionally demonstrate equivocal or discordant p57 and β-hCG immunostaining due to biological variability, such as genetic mosaicism, early gestational age, and altered trophoblastic differentiation, as well as technical and interpretative factors related to tissue fixation, antibody performance, and internal control assessment. These factors can result in focal, weak, or heterogeneous staining patterns, thereby justifying their classification as equivocal or discordant cases despite morphologic features consistent with complete hydatidiform mole. Nonetheless, interpretative pitfalls exist: focal staining in CHM amniotic tissue due to mosaicism, retention of maternal chromosome 11, or rare biparental CHM cases may yield p57 positivity despite CHM status [28]. Similarly, rare PHM cases with loss of maternal chromosome 11 may falsely appear p57 negative [28]. These caveats underscore the need for a combined algorithmic approach in which genotyping remains the essential gold standard where immunohistochemical findings are ambiguous.

### 3.3. Features of Ki-67, E-Cadherin, and β-hCG Immunoexpression

Beyond p57, proliferation and differentiation markers such as Ki-67, β-hCG, and E-cadherin contributed with valuable supplemental diagnostic refinement in this study. We observed a gradient of Ki-67 immunostaining intensity, with CHMs inclining toward stronger proliferative indices compared to PHMs and HAs, thus indicating more aggressive trophoblastic activity. According to our results, Ki-67 supports diagnostic and prognostic assessments but should not be regarded as a definitive marker.

A significant meta-analysis shows higher Ki-67 labeling in CHMs than in PHMs or hydropic abortions, particularly in villous cytotrophoblasts, so Ki-67 is a useful adjunct when p57 and morphology are inconclusive. Absolute cut-offs vary between studies and laboratories: Ki-67 is best used qualitatively or with lab-validated thresholds [23].

Similarly, β-hCG expression—highest in CHMs, less in PHMs, and lowest in HAs—reflects the underlying pathophysiology, where androgenetic genomes drive exaggerated syncytiotrophoblast proliferation and hCG secretion. Immunohistochemically, β-hCG is reserved for confirming trophoblastic differentiation (syncytiotrophoblast) or when the tumor vs. non-tumor trophoblastic lineage is in question. It cannot reliably discriminate CHMs from PHMs [14,29].

E-cadherin, a cell adhesion molecule variably expressed, exhibited highest immunoscores in HAs, intermediate in PHMs, and lowest in CHMs, potentially mirroring varying epithelial differentiation or cellular cohesion among these entities. Loss or reduced membranous E-cadherin may indicate epithelial–mesenchymal transition/invasive potential and can help separate molar tissue from hydropic abortus in some series [2]. In our context, it can be used when additional evidence distinguishing molar from non-molar hydropic tissue is needed [2]. Thus, despite its biological importance, E-cadherin has limited applicability in diagnosis, which resonates with the results identified in other studies [30,31].

Taken together, these findings resonate with earlier studies combining IHC with ploidy analysis or DNA cytometry, such as the study of Osterheld et al., which demonstrated that dual-modal assessment (p57 and DNA content) in 111 first-trimester conceptions significantly improved differentiation between HAs, PHMs, and CHMs, particularly where ploidy provided context to ambiguous immunohistochemical results [32]. Our retrospective dataset similarly illustrates that while histology lays the initial groundwork, it must be supplemented by an algorithmic histopathology–IHC–genotyping approach to reduce diagnostic error and interobserver variability [33]. Moreover, our findings suggest that p57 and β-hCG are the most dependable diagnostic markers, while Ki-67 offers additional insight into proliferative activity. Although it reveals pathophysiological differences, E-cadherin has limited diagnostic utility.

On the same note, other studies confirm that different immunohistochemical markers (Ki-67, E-cadherin, Twist1, p53, inhibin-α, and combinations) have been studied as adjuncts, but none replace genotyping or p57 for classification, presenting inconsistent diagnostic utility [2,16].

### 3.4. Enhancing Diagnostic Accuracy and Clinical Management in Trophoblastic Disease

Morphology offers relative information in the diagnosis of molar pregnancies, requiring the corroboration with clinical, paraclinical, and imagistic data, as well as a series of ancillary techniques. The histopathological diagnosis of HMs continues to be negatively influenced by interobserver variability, an algorithmic histopathology–IHC approach completed by genotyping, refining the accuracy of the histopathological examination.

In a clinical context, accurate distinction among CHMs, PHMs, and HAs is not merely academic: it guides postpartum surveillance, informs risk stratification for persistent gestational trophoblastic disease (higher in CHMs and intermediate in PHMs), and facilitates appropriate patient counseling. Therefore, despite its limitations, IHC, especially p57 assessment, serves as a practical and cost-effective surrogate for maternal genomic presence. Still, the integration of molecular genotyping remains critical in ambiguous or high-stakes cases, most notably given rare exceptions and familial biparental CHMs.

In sum, our study reaffirms that morphology, together with IHC, augmented by proliferation/differentiation profiles and backed by molecular genotyping where needed, constitutes a best-practice paradigm for hydatidiform mole diagnosis. This integrated framework promises to enhance reproducibility, refine diagnostic accuracy, and optimize patient management strategies in trophoblastic pathology.

### 3.5. Diagnostic Algorithmic Approach for Hydropic Villous Pathology (CHM, PHM, and HA)

The development of a diagnostic algorithm centered primarily on immunohistochemical evaluation of p57, β-hCG, E-cadherin, and Ki-67 is justified by the complementary biological and diagnostic information provided by these markers. p57 serves as a critical discriminator of androgenetic conceptions, as its expression reflects the presence of maternally derived genomic material, making it particularly valuable in the distinction between complete and partial moles. β-hCG highlights trophoblastic activity, supporting assessment of disease activity and correlating with clinical behavior. E-cadherin aids in evaluating cellular cohesion and trophoblastic differentiation, helping to distinguish molar from non-molar gestations and to exclude mimickers. Ki-67 provides an objective measure of proliferative activity, contributing to risk stratification and reinforcing morphologic impressions.

**Figure 7:**
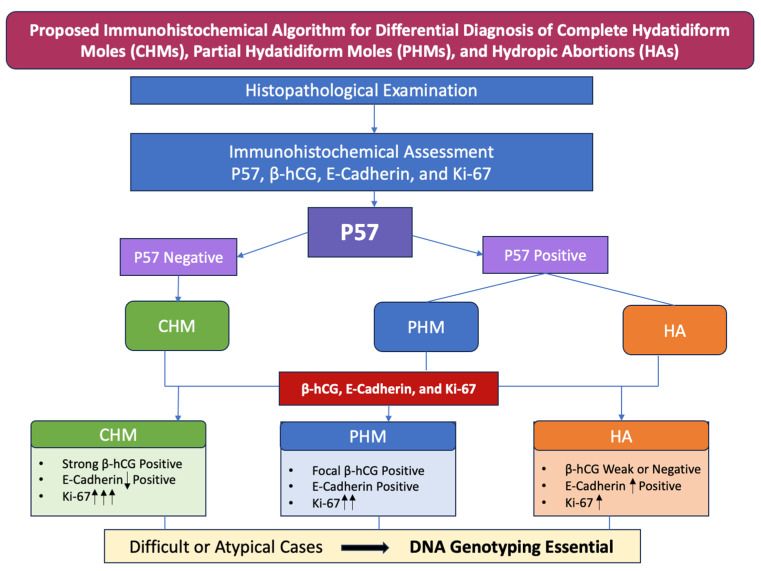
Proposed immunohistochemical algorithm for differential diagnosis of complete hydatidiform moles (CHMs), partial hydatidiform moles (PHMs), and hydropic abortions (HAs). E-Cadherin: ↓ Positive—reduced membranous immunoexpression; **↑** Positive—high membranous immunoexpression; Ki-67: **↑**—weak immunoexpression; **↑↑**—moderate immunoexpression; **↑↑↑**—strong immunoexpression.

Within the algorithm (Figure 7), these markers are applied in a stepwise fashion following histologic evaluation, allowing most cases to be classified with high confidence while reducing subjectivity. Genotyping becomes mandatory in scenarios where immunohistochemical results are inconclusive or discordant, such as equivocal or heterogeneous p57 staining, unusual morphologic features, or when distinguishing partial moles from non-molar gestations with overlapping immunoprofiles. In these settings, genotyping provides definitive clarification of parental genomic contributions, ensuring diagnostic accuracy and appropriate clinical management.

Such an immunohistochemical-driven, algorithmic approach is particularly valuable in pathology departments where access to molecular genotyping is limited or not routinely available. By relying on widely accessible, cost-effective immunohistochemical markers, the algorithm enables accurate classification in the majority of cases and supports timely diagnostic decision making. In this context, genotyping can be judiciously reserved for diagnostically challenging or unresolved cases, optimizing resource utilization while maintaining a high standard of diagnostic precision.

## 4. Materials and Methods

### 4.1. Patients

Our retrospective study analyzed a group including 64 consecutive patients, ranging in age from 17 to 36, diagnosed with placental pathological conditions between 2010 and 2024 at the “Elena Doamna” Obstetrics and Gynecology Clinical Hospital in Iași, Romania. The study protocol was approved by the Ethics Committee of “Elena Doamna” Clinical Hospital of Obstetrics and Gynecology (Nr. 207/13 January 2025) based on written informed consent for the use of archived biological material for research.

The study group comprised 10 hydropic abortions (HAs), 38 partial hydatidiform moles (PHMs), and 16 complete hydatidiform moles (CHMs), as determined by the original pathology reports.

The consecutive cases with products of conception (POC) obtained from uterine evacuation (dilatation and curettage) were analyzed in the retrospective study based on the following inclusion criteria: clinically suspected and histologically diagnosed complete and partial HMs, clinically and histologically diagnosed non-molar abortion (spontaneous or missed abortion), histopathological specimens with adequate tissue preservation and representative chorionic villi available for IHC evaluation, and cases with complete clinical and pathological data, including maternal age, gestational age, clinical presentation, and ultrasound findings (when available).

The exclusion criteria referred to inadequate or poorly preserved specimens, including autolyzed tissue, specimens with extensive hemorrhage, necrosis, crush artifacts, or those with insufficient chorionic villi for reliable IHC analysis.

Clinico-demographical data, including age, parity, history of previous miscarriages, gestational age at diagnosis, dynamic β-hCG levels, symptomatology, ultrasound findings, and associated medical conditions, were available for all cases.

The patients’ ages ranged from 17 to 42 years, but after applying the exclusion criteria, the age range of consecutive cases included in the study was 17–36 years.

Among our selected cases, the average ages for CHMs were around 34–36 years old (10 out of 16 cases), with 2 cases under 20 years, and for PHMs were around 25–35 years old (24 out of 38 cases), with 3 cases under 20 years old; the average ages for HA were around 28–32 years old (6 out of 10 cases). With the exception of 6 patients under 20 years old, diagnosed with gestational trophoblastic disease (CHM or PHM) or HA, and 5 patients between 24 and 30 years old, diagnosed with molar pregnancy or HA, who were nulliparous, the rest of the patients in the study were primiparous or multiparous. No miscarriages and no significant family pregnancy-associated medical history were noted for the selected cases.

For all study cases, the main symptomatology was vaginal bleeding, sometimes associated with pelvic pain. Among the associated clinical conditions, two 20-year-old patients, diagnosed with CHMs, had associated SARS-CoV-2 infection and cystic ovaries, respectively.

All cases with CHMs presented initial β-hCG values > 100,000 mIU/mL, reaching maximum values of >600,000 mIU/mL. PHM presented β-hCG values between 20,000 and 150,000 mIU/mL, and for HA cases, β-hCG values varied between 5000 and 50,000 mIU/mL, with the variability of values being correlated with the gestational age.

However, these demographic and clinico-pathological features were not incorporated into the statistical analysis because they were not directly relevant to the aim of the study. The IHC markers evaluated (p57, E-cadherin, β-hCG, and Ki-67) are interpreted at the tissue level and are not known to be significantly influenced by obstetric history in a way that would alter their diagnostic utility. Moreover, the exploratory character of our study, based on a small number of patients enrolled, as well as the diversity of IHC expression of the markers did not allow for specific statistical correlation tests.

Two independent pathologists, blinded to clinical data, reviewed hematoxylin and eosin (H&E)-stained sections and grouped each case by its defining morphological features: CHMs exhibited villous hydropic swelling with central cistern formation, circumferential trophoblastic hyperplasia with diffuse marked atypia, and trophoblastic inclusions; PHMs showed focal trophoblastic hyperplasia, a dimorphic population of hydropic and normal villi with scalloping, prominent stromal trophoblastic inclusions, and mild atypia; HA was characterized by villous edema without trophoblastic hyperplasia.

Formalin-fixed, paraffin-embedded placental specimens corresponding to the patients were extracted from the archives of the Department of Pathology at the “Elena Doamna” Obstetrics and Gynecology Clinical Hospital in Iași, Romania, to perform the IHC exam.

### 4.2. Tissue Preparation for Immunohistochemical Staining

The formalin-fixed, paraffin-embedded organ samples from all cases were sectioned at 4 µm thicknesses and mounted on positively charged glass slides. Sections were deparaffinized in xylene, rehydrated through graded alcohols, and subjected to antigen retrieval according to the manufacturer’s protocols. IHC staining was performed using an automated platform (LEICA BOND-MAX™, Leica Biosystems, Nussloch, Germany) to ensure reproducibility and standardization of staining conditions.

The following primary antibodies were employed: rabbit polyclonal anti-p57 (clone STJ16100411, dilution 1:300, St John’s Laboratory, London, UK), mouse monoclonal anti-Ki-67 (clone NCL-L-Ki67-MM1, dilution 1:100, Leica Novocastra, Nussloch, Germany), rabbit polyclonal anti-hCG (ab9376, dilution 1:100, Abcam, Cambridge, UK), and mouse monoclonal anti-human E-cadherin (clone NCH-38, ready-to-use, Dako, Glostrup, Denmark). All antibodies were incubated according to optimized protocols, followed by detection using a polymer-based detection system and visualization with 3,3′-diaminobenzidine (DAB). Hematoxylin was used as the counterstain.

The same two independent pathologists, blinded to clinical data, assessed all slides for a semiquantitative evaluation of the biomarkers’ immunoexpression. Discordant results were reviewed jointly to reach consensus.

### 4.3. Scoring of p57, Ki-67, β-hCG, and E-Cadherin Immunoexpression

The semiquantitative assessment was manually performed by two pathologists using specific scoring systems validated in the mainstream for each of the analyzed markers. Scoring differences due to interobserver variability were discussed in order to reach an agreement.

For p57, the nuclear staining pattern (positive/absent) in villous cytotrophoblast and villous stromal cells was assessed by counting 100 positive cells on 3–5 fields at high magnification (×400), with extravillous trophoblasts and maternal decidua serving as internal positive controls [22]. p57 expression was considered adequately negative when villous stromal cells and cytotrophoblasts showed no staining, provided that internal positive controls were present (for example, maternal decidua and/or intermediate trophoblasts showing nuclear p57 expression). p57 immunoreactivity was considered positive when these cell types exhibited widespread or diffuse nuclear staining [22]. According to the literature, p57 immunostain was considered equivocal when nuclear staining was focally present in both villous stromal cells and cytotrophoblast, involving at least 10% but fewer than 50% of villi in the stained section. The immunoexpression was classified as discordant when mixed patterns of positivity and negativity were observed between villous stromal cells and cytotrophoblast within the same villi, including cases in which one cell type was positive while the other was negative [22].

For Ki-67, nuclear labeling indices were evaluated in villous cytotrophoblasts and stromal cells by counting 100 positive cells on each slide in “hot spots” (highest-staining areas) at high magnification (×400). The staining intensity was semiquantitatively classified as follows: 0 (no positive cells), low (+; ≤25% positive cells), moderate (++; 26–50% positive cells), and high (+++; >50% positive cells) (Table 6) [2].

β-hCG expression was evaluated at the cytoplasmic level in villous trophoblast using an adapted semiquantitative immunoscore [13] (Table 7), considering both the intensity (I) of staining and the percentage of positively stained cells (P), calculated by multiplying the two values (I × P), yielding a final score from 0 to 12 [34]. Accordingly, 100 positive cells on 3–5 fields at high magnification (×400) were counted. In this regard, staining intensity (I) was rated on a 0–3 scale as negative, weak, moderate, or strong. The proportion of positive cells (P) was evaluated on a 0–4 scale as negative, <25%, 26–50%, 51–75%, or >75%. Staining expression was then categorized as negative (0), very weak (1, 2), weak (3, 4), moderate (6, 8), and strong (9, 12) [34].

E-cadherin expression was identified by membranous staining in villous cytotrophoblast, which was used as the criterion for a positive result. Expression was evaluated semiquantitatively based on staining intensity and the percentage of positive cells (Table 7). The E-cadherin immunoreactivity score was determined by multiplying the percentage of positive cells by the staining intensity. To estimate the percentage of positive cells, approximately 100 cells per slide on 3–5 fields at high magnification (×400) were counted and scored as follows: 0 = <5% positive cells; 1 = 5–25%; 2 = 25–50%; 3 = 50–75%; and 4 = >75%. Staining intensity was graded on a scale of 0 to 3, where 0 = negative, 1 = weak, 2 = moderate, and 3 = strong. The E-cadherin immunoreactivity score was divided as follows: 0—negative; 1, 2—very low; 3, 4—low; 6, 8—moderate; and 9, 12—high [2,34].

### 4.4. Statistical Analysis

All statistical analyses were performed to evaluate the diagnostic performance of the IHC markers in differentiating complete hydatidiform moles (CHMs), partial hydatidiform moles (PHMs), and hydropic abortions (HAs). Data were analyzed using IBM SPSS Statistics V. 30 (IBM Corporation, Armonk, NY, USA). Descriptive statistics (frequency, percentage, median, and interquartile range) were calculated for each marker. For categorical data (p57 positivity), comparisons among CHMs, PHMs, and HAs were performed using the Chi-square (χ^2^) test; Fisher’s exact test was applied when expected cell counts were <5. For ordinal and semiquantitative variables (Ki-67, β-hCG, and E-cadherin), the Kruskal–Wallis test was used to assess differences among the three groups, followed by Mann–Whitney U tests with Bonferroni correction for pairwise comparisons. Diagnostic accuracy of individual markers was evaluated by calculating the sensitivity, specificity, positive predictive value (PPV), and negative predictive value (NPV). Receiver operating characteristic (ROC) curve analysis was applied for Ki-67, β-hCG, and E-cadherin immunoscores, with area-under-the-curve (AUC) values reported. Statistical significance was defined as *p* < 0.05.

In order to perform the ROC/AUC study used for Ki-67, E-cadherin, β-hCG, as the gold standard for diagnosing and classifying hydatidiform moles (CHM vs. PHM vs. non-molar hydropic abortion), we consider the histopathologic evaluation combined with p57 IHC. The gold standard considered was consistent with published studies, which state that if genotyping is not available, the evidence-based practical “gold standard” used for ROC studies is a composite reference standard of histopathology, evaluated by 2 or more experienced gynecologic pathologists and p57 (p57KIP2) immunohistochemistry [19,20].

To quantify each marker’s discriminative ability between CHMs and non-CHMs (PHMs and HAs), receiver operating characteristic (ROC) curve analysis was performed. ROC curves were generated by plotting sensitivity (true positive rate) against 1 − specificity (false positive rate) across all possible thresholds. The area under the ROC curve (AUC) was calculated to provide a summary measure of diagnostic accuracy. AUC values were interpreted as follows: excellent (≥0.90), good (0.80–0.89), fair (0.70–0.79), poor (0.60–0.69), and no discrimination (0.50). Optimal cut-off values were determined according to the Youden index (J = sensitivity + specificity − 1) where applicable.

## 5. Conclusions

Our study sought to identify a practical alternative to molecular genotyping by using p57, Ki-67, β-hCG, and E-cadherin immunoreactivity to mainly differentiate PHMs from non-molar pregnancies, particularly HA cases. Unlike molecular methods, which are rarely used in routine practice due to their expense and lengthy processing time, this immunohistochemical combination offers a more efficient and cost-effective option, especially in resource-limited settings. In summary, ancillary techniques can significantly enhance the diagnosis of molar pregnancies. p57 and β-hCG are the most dependable diagnostic markers, while the combined Ki-67 and E-cadherin approach provides a reliable alternative for the challenging differential diagnosis between PHMs and HAs. These considerations are further supported by our proposed diagnostic algorithm, which relies predominantly on immunohistochemical assessment of HMs and HAs and may constitute an accurate and efficient diagnostic strategy in the evaluation of molar pathology.

## Figures and Tables

**Table 1:** The immunoexpression of p57 in CHMs, PHMs, and HAs.

Antibody	CHM Negative	CHM Discordant Positive	CHM Positive Equivocal	PHM Negative	PHM Positive	HA Negative	HA Positive
p57	9 (57%)	3 (18%)	4 (25%)	-	38 (100%)	-	10 (100%)

**Table 2:** The immunoexpression of Ki-67 in CHMs, PHMs, and HAs.

Antibody	CHM Negative	CHM Positive	CHM Positive Equivocal/ Discordant	PHM Negative	PHM Positive	HA Negative	HA Positive
Ki-67	-	2 (12.5%) weak	-	-	21 (57.8%) weak	-	10 (100%) weak
		8 (50%) moderate			9 (23.68%) moderate		
		6 (37.5%) strong			4 (10.52%) strong		

**Table 3:** The immunoexpression of β-hCG in CHMs, PHMs, and HAs.

Antibody	CHM Negative	CHM Positive	CHM Positive Equivocal	PHM Negative	PHM Positive	HA Negative	HA Positive
β-hCG	-	10 (62.5%) 12	1 (20%) 2 very weak	-	36 (94.73%) 6	4 (40%) 0	2 (20%) 2
		5 (31.25%) 9			2 (5.26%) 4		4 (40%) 1

**Table 4:** The immunoexpression of E-cadherin in CHMs, PHMs, and HAs.

Antibody	CHM Negative	CHM Positive	CHM Positive Equivocal	PHM Negative	PHM Positive	HA Negative	HA Positive
E-cadherin	-	2 (12.5%) 9	-	-	2 (5.26%) 12	-	7 (70%) 12
		7 (43.75%) 6			8 (21.05%) 8		2 (20%) 9
		7 (43.75%) 2			22 (57.89%) 6		1 (10%) 8
					6 (15.78%) 4		

**Table 5:** Statistical analysis and diagnostic performance of immunohistochemical markers in HMs and HAs.

Marker	Statistical Tests (Overall + Pairwise)	ROC AUC	Sensitivity	Specificity	PPV	NPV	Interpretation
p57	χ^2^ = 26.94, *p* = 2.1 × 10^−7^; Fisher’s exact *p* < 2 × 10^−7^ (CHM vs. PHM + HA)	-	56–75% (depending on equivocal staining)	100%	100%	87–94%	Most specific marker for CHM; negativity strongly supports diagnosis. Equivocal staining reduces sensitivity: genotyping recommended in indeterminate cases.
Ki-67	χ^2^ = 21.1, *p* = 0.0003; Kruskal–Wallis *p* < 4 × 10^−5^. Pairwise MWU: CHM vs. PHM *p* = 0.0009, CHM vs. HA *p* < 0.0001, and PHM vs. HA *p* = 0.027	0.81	87.5%	71.1%	51.9%	94.1%	Reflects proliferation gradient (CHM ≫ PHM > HA). Good sensitivity but overlap with PHM lowers specificity.
β-hCG	χ^2^ = 121.1, *p* = 3.8 × 10^−20^; Kruskal–Wallis *p* < 4.3 × 10^−12^. Pairwise MWU all highly significant (*p* < 0.001)	0.95	93.8%	100%	100%	98%	Best overall discriminator. CHM shows very high expression, PHM intermediate, and HA minimal/absent.
E-cadherin	χ^2^ = 67.7, *p* = 1.3 × 10^−10^; Kruskal–Wallis *p* = 1.1 × 10^−6^. Pairwise MWU: CHM vs. HA *p* < 0.0002, PHM vs. HA *p* < 0.00001, and CHM vs. PHM *p* = 0.06	0.25	87.5%	41.7%	33.3%	90.9%	Downregulated in CHM but retained in HA. Biologically relevant but limited diagnostic utility due to poor specificity.

**Table 6:** Scoring system of Ki-67 immunostaining.

Staining Intensity	Score
0—no positive cells	Negative
+—≤25% positive cells	Low
++—26–50% positive cells	Moderate
+++—>50% positive cells	High

**Table 7:** Scoring systems of β-hCG and E-cadherin immunostaining.

Staining Intensity (I)	Percentage of Positive Cells (P)
0—Negative 1—Weak intracytoplasmic staining 2—Moderate intracytoplasmic staining 3—Strong intracytoplasmic staining	0—Negative (<5%) 1—5–25% 2—25–50% 3—50–75% 4—>75%

## Data Availability

The original contributions presented in this study are included in the article. Further inquiries can be directed to the corresponding author.
